# Supplementary material for: A set of multi-entry identification keys to African frugivorous flies (Diptera, Tephritidae)
Source: Zookeys. 2014 Jul 24;(428):97–108. doi: 10.3897/zookeys.428.7366 (PMC4143993; doi:10.3897/zookeys.428.7366)
Supplement: Supplementary material 4 — Key to Capparimyia [file zookeys-428-097-s004.zip › SF4_ZooKeys_key to Capparimyia/lucid_player/help/help/un-choosing.htm]

Un-choosing and changing chosen states


# Un-choosing and changing chosen states

Chosen states in Lucid3 are checked with a tick in both the *Features
Available* and *Features Chosen*
lists. To remove a previously chosen state, simply uncheck its checkbox
in either list. Note that unchecked states remain in *Features
Chosen* until the key is restarted - this allows them to be easily
rechosen if necessary.

To change the value of a chosen numeric feature, click on its edit box
in *Features Chosen* then change
the values entered in the *Numerics*
dialog box. To unchoose the feature completely, remove any values from
the dialog box and click OK.
